# Text-Based Program Addressing the Mental Health of Soon-to-be and New Fathers (SMS4dads): Protocol for a Randomized Controlled Trial

**DOI:** 10.2196/resprot.8368

**Published:** 2018-02-06

**Authors:** Richard Fletcher, Chris May, John Attia, Craig Franklin Garfield, Geoff Skinner

**Affiliations:** ^1^ Family Action Centre Faculty of Health and Medicine University of Newcastle Callaghan Australia; ^2^ Hunter Medical Research Institute Faculty of Health and Medicine University of Newcastle Callaghan Australia; ^3^ Department of Pediatrics Feinberg School of Medicine Northwestern University Chicago, IL United States; ^4^ School of Electrical Engineering and Computing Faculty of Engineering and Built Environment University of Newcastle Callaghan Australia

**Keywords:** perinatal, fathers, online intervention, randomized controlled trial, mental health

## Abstract

**Background:**

Recent estimates indicating that approximately 10% of fathers experience Paternal Perinatal Depression (PPND) and the increasing evidence of the impact of PPND on child development suggest that identifying and assisting distressed fathers is justified on public health grounds. However, addressing new fathers’ mental health needs requires overcoming men’s infrequent contact with perinatal health services and their reluctance to seek help. Text-based interventions delivering information and support have the potential to reach such groups in order to reduce the impact of paternal perinatal distress and to improve the wellbeing of their children. While programs utilising mobile phone technology have been developed for mothers, fathers have not been targeted. Since text messages can be delivered to individual mobile phones to be accessed at a time that is convenient, it may provide a novel channel for engaging with “hard-to-reach” fathers in a critical period of their parenting.

**Objective:**

The study will test the efficacy of SMS4dads, a text messaging program designed specifically for fathers including embedded links to online information and regular invitations (Mood Tracker) to monitor their mood, in order to reduce self-reported depression, anxiety and stress over the perinatal period.

**Methods:**

A total of 800 fathers-to-be or new fathers from within Australia will be recruited via the SMS4dads website and randomized to the intervention or control arm. The intervention arm will receive 14 texts per month addressing fathers’ physical and mental health, their relationship with their child, and coparenting with their partner. The control, SMS4health, delivers generic health promotion messages twice per month. Messages are timed according to the babies’ expected or actual date of birth and fathers can enroll from 16 weeks into the pregnancy until their infant is 12 weeks of age. Participants complete questionnaires assessing depression, anxiety, stress, and alcohol at baseline and 24 weeks postenrolment. Measures of coparenting and parenting confidence are also completed at baseline and 24 weeks for postbirth enrolments.

**Results:**

Participant were recruited between October 2016 and September 2017. Follow-up data collection has commenced and will be completed in March 2018 with results expected in June 2018.

**Conclusions:**

This study’s findings will assess the efficacy of a novel text-based program specifically targeting fathers in the perinatal period to improve their depression, anxiety and distress symptoms, coparenting quality, and parenting self-confidence.

**Trial Registration:**

Australian New Zealand Clinical Trials Registry ACTRN12616000261415; https://www.anzctr.org.au/ Trial/Registration/TrialReview.aspx?id=370085 (Archived by WebCite at http://www.webcitation.org/6wav55wII).

## Introduction

The period surrounding childbirth represents a vulnerable time for families where both women and men may be at increased risk of mental health disorders. However, while maternal perinatal screening and treatment protocols have been introduced within Australia, Paternal Perinatal Depression (PPND) has been largely overlooked [1]. Recent estimates indicating that 9% to 10% of fathers experience PPND [2,3] and the increasing evidence of the impact of PPND on child development [4,5] suggest that identifying and assisting distressed fathers is justified on public health grounds. In similar fashion, high levels of paternal anxiety and stress have also been found to be harmful to children’s development [6-8]. However, addressing new fathers’ mental health needs requires overcoming men’s infrequent contact with perinatal health services and their reluctance to seek help [9,10].

While anxiety, stress and depression are recognized as related negative emotional states, paternal depression has figured most prominently in the published research on fathers’ mental health in the perinatal period. PPND can impair the fathers’ relationship with his infant and reduce his ability to effectively coparent. Observational studies of depressed fathers have found decreased levels of warmth, sensitivity, synchrony engagement, and positive involvement and increased levels of criticism, hostility, harshness, intrusiveness, withdrawal, and control [11]. For example, fathers of one year olds who had experienced a major depressive episode in the previous year were only half as likely to read to their infants but four times more likely to spank them [12].

The detrimental impact of PPND on fathers' mental wellbeing and on infant development is well documented. Preschoolers whose fathers reported symptoms of depression in the first year had twice the risk of behavioral and emotional problems compared to children of nondepressed fathers [13]. When the children were assessed at 7 years of age, those whose fathers had been depressed following their birth were almost twice as likely to have a psychiatric disorder compared to those of nondepressed fathers [4]. In both studies, findings were consistent after adjusting for maternal depression and paternal educational level. In addition, analysis of an Australian cohort found highly elevated behavior problems in preschool children whose fathers had shown depressive symptoms in their first year [5].

PPND, expressed through hostility and negative comments, is also likely to impact on the relationship between the parents, especially when combined with alcohol dependence [14,15]. Couple conflict and lack of partner support has been linked to maternal postnatal depression [16,17], and marital conflict may help to explain the relationship between postnatal depression in either mothers' or fathers' and child outcomes [18,19].

Given the deleterious effects of PPND on a father's own health, his parenting and coparenting relationship, and the possible ongoing impairment of his child's development, the need to identify and treat or support distressed fathers is clear. Moreover, health service costs of PPND in Australia, using 2012 figures, have been estimated at $17.97 million with an added cost of $223.75 million due to lost productivity [20]. While calls for fathers to be assessed for mood disorders and offered information, support, and counselling during the perinatal period are common, the service response has been absent [21].

Addressing PPND will require overcoming several barriers. Work demands, for example, prevent most fathers participating in perinatal clinic visits with their partner [9]. Those fathers attending antenatal preparation classes or the birth may feel ignored by professionals and feel that they have few opportunities to raise their concerns [22,23]. As well, fathers may have little understanding of perinatal depression in general and may not easily recognize their own symptoms of depression [24,25]. Attempts to engage fathers in early intervention programs have had little success and fathers are badged as “hard to reach” [26,27].

Mobile phone technology may be feasible in identifying distressed fathers and offering support as ownership among Australian adults is high (94%) [28]. Fathers regularly use the Internet to source parenting information [29,30]; however, linking fathers to Web-based parenting resources remains problematic. The low recruitment and engagement of fathers found in face-to-face parenting programs [28] appears to be replicated in online parenting programs [31,32].

Text messaging has been shown to influence health-related knowledge and beliefs among mothers. Text4baby, a United States–based program sending short message service messages to mothers through the perinatal period, has found that 75% of users reported text messaging had given them important medical information. A randomized controlled trial involving 90 Text4baby users found that text messaging was associated with positive alterations in targeted health beliefs [33]. Health behaviors relating to smoking, weight loss, physical activity and the management of diabetes have also shown improvement in response to text messaging [34,35]. Text messaging could therefore provide a novel avenue for delivering support and parenting-related information to fathers during their transition to fatherhood.

While texts, which are delivered at no cost to participants, are able to convey information relevant to a father’s role during pregnancy and postbirth, they have limited capacity to engage fathers in help seeking. Self-monitoring to increase participants’ awareness of their mood has been incorporated into web and mobile phone programs targeting depression [36,37] with embedded links to websites for further information, education, or support. Providing links within messages to websites that are relevant to fathers' concerns and including interactive text messages inviting mood self-monitoring (Mood Tracking) may encourage fathers to seek out assistance for parenting difficulties and for personal distress.

Text-based interventions delivering information and support have the potential to reduce the impact of perinatal distress in parents and improve the wellbeing of their children. While programs utilising mobile phone technology have been developed for mothers’ mental health [38] fathers have not been targeted. The study proposed here will test the efficacy of a program incorporating brief text messages designed specifically for fathers including embedded links to online information and support and regular invitations (Mood Tracker) to monitor their mood (see Multimedia Appendices 1, 2, and 3). Since text messaging is relatively inexpensive and can be accessed across rural and urban areas at a time that is convenient, it may provide a novel channel for reaching “hard-to-reach” fathers in a critical period of their parenting. SMS4dads may offer a low-cost model for improving the wellbeing of families through supporting the male partner in the parenting team.

The aim is to conduct the first randomized controlled trial of a text-based intervention, SMS4dads, which targets fathers’ mental health during the perinatal period. The efficacy of the SMS4dads program in reducing depression, anxiety, and stress symptoms among new fathers will be assessed by comparing intervention outcomes with those of a sham program, SMS4health, offering generic health promotion messages. Secondary aims are to assess the effect of SMS4dads on the fathers’ coparenting, parenting self-confidence, and use of alcohol.

### Hypotheses

It is predicted that fathers receiving the SMS4dads program will show lower levels of depression, anxiety, and distress at 24 weeks postbaseline than fathers receiving the SMS4health program. In addition, fathers’ coparenting and parenting self-confidence are predicted to be greater and use of alcohol to be less among those receiving SMS4dads than among those receiving SMS4health.

## Methods

**Figure 1:**
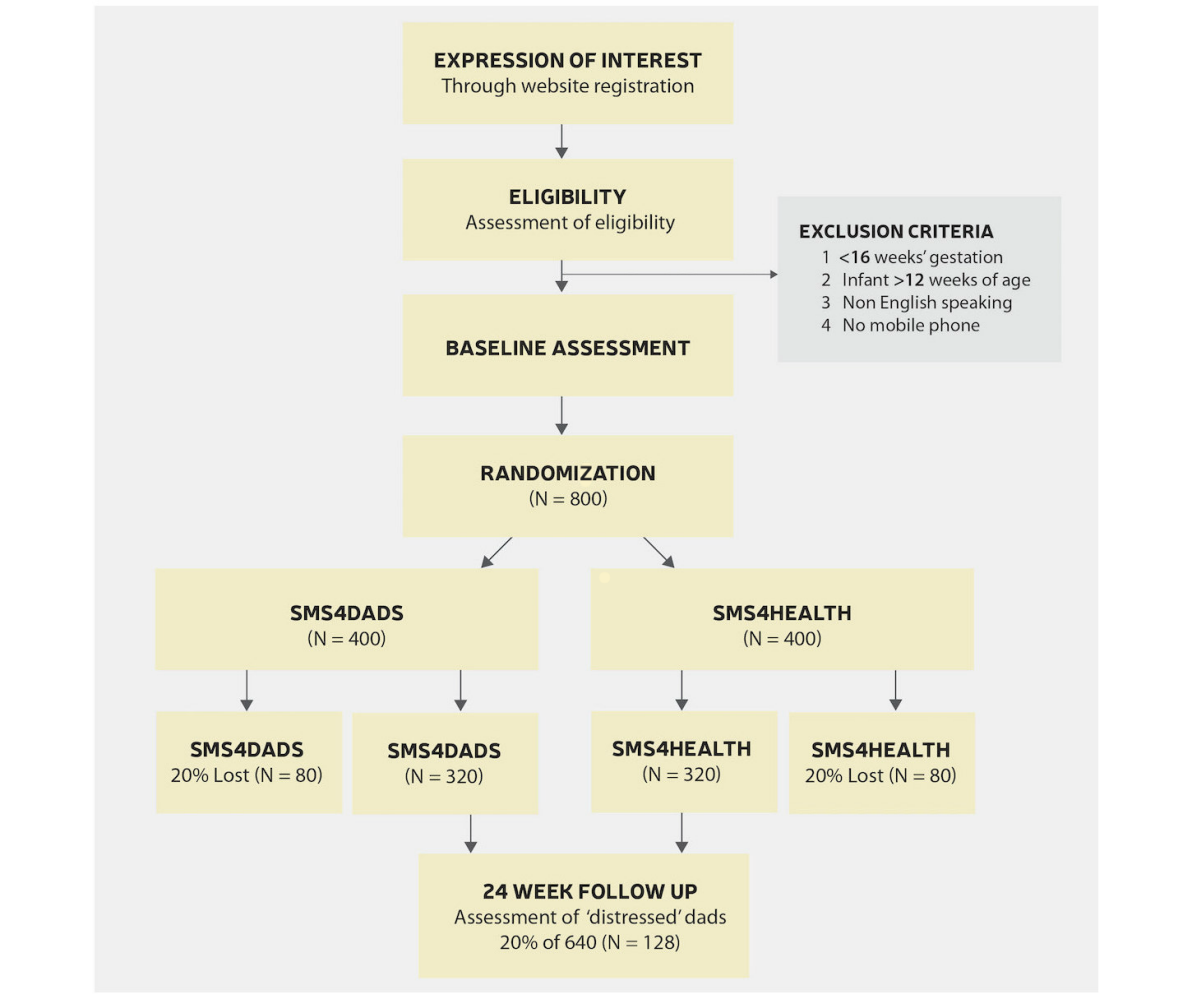
Participant flow through the trial.

This randomized controlled trial is designed to assess the efficacy of a text-based intervention targeting mental health in new fathers. Participating fathers will be randomly allocated to receive either the SMS4dads or SMS4health. A summary of participant flow though the trial is provided in Figure 1. This study received ethics approval from The University of Newcastle Human Research Ethics Committee on June 16 2016, approval number H-2016-0055.

### Study Sample and Procedure

A total of 800 fathers-to-be or new fathers from within Australia will be recruited. Participants will be recruited through the SMS4dads website [36], which houses a brief introductory video explaining the SMS4dads program and information and enrolment pages. Fathers will be made aware of the Web page and the project through social media, through flyers distributed by health staff in contact with parents, and through mainstream media outlets. Interested fathers are provided with email and phone contact to enable further questions about the study to be answered.

### Inclusion Criteria

Participants will be included in the trial if they indicate having a partner who is more than 16 weeks pregnant or their infant is less than 12 weeks of age, have a mobile phone capable of receiving text messages, and they can read and understand English. There is no cost involved in receiving the text messages but access to the internet is required to respond to the Mood Tracker texts and to access the links provided to websites in the messages.

### Screening and Baseline

Expecting and new fathers will register for participation through the project website where an information statement and other materials about the project are readily available [39]. Fathers wishing to enrol can click on the “Join Up” tab located on the front page of the SMS4dads website, which will take them to the registration pages. The first registration pages assess eligibility and information for messaging purposes including their infant’s expected or actual date of birth, full name, email address, and phone number. For demographic purposes, they will be asked for their age, postal address, Indigenous status, and a question about their socioeconomic position [37]. When completing consent questions fathers will be asked to confirm that they have read the information statement and that they have no further questions about the project. They will also be asked if they would like to be informed of study outcomes, if they would be willing to participate in phone interviews about their experience of participation, and whether they would be prepared to pass an information package onto their partner. When fathers have answered these questions they will be asked to respond to four questionnaires (53 items) detailed in the following section on study measures. All questions mandate a response but fathers can report that they have no email address and that they do not wish to supply data about their socioeconomic position.

### Follow-Up Assessments

Fathers are also asked to repeat the questionnaires 24 weeks after receiving their first message.

### Randomization

Randomization was performed using a custom-built Web-based module attached to the online data collection forms. The actual randomization process was based on a random number generator with a random seed, and using random permuted blocks of 4 or 6. Allocation was 1:1 in the intervention or control arm as new users registered on the site. The Web modules were built using C#.Net (with a Visual studio integrated development environment) and a Microsoft SQL Server backend to store the generated random numbers.

From an architectural perspective, proper separation of code design principles was followed as the complete randomization feature was implemented as a contained application programming interface (API) module. The API module was only accessible from a software Application with the required granted permissions, through calls within the requesting Applications business and data access layer. This also allowed the module to be maintained without effecting the source Web application consuming its resources

### Sample Size

Sample size was based on the primary outcome of Depression Anxiety Stress Scales (DASS-21) [40] values in the intervention and control arms at 24 weeks, adjusting for baseline values. We assume an effect size of 0.5 SD Units (Cohen’s *d*) among those “distressed” fathers who report Moderate, Severe or Extremely Severe scores on the total DASS-21 (≥ 23). Aiming for power of 80%, significance level of 5%, 1:1 ratio of allocation to control and intervention, we estimate a sample size of 64 “distressed” fathers will be required in each arm of the trial. Assuming 20% of the sample recruited will be distressed (based on feasibility study data) we will require 320 fathers in each arm giving a total sample of 640 fathers. Allowing 20% for attrition and loss to follow-up, 800 fathers in total will be required to be recruited.

### Program Content, Tone, and Design

The program content includes an established corpus of relationship-focused messages developed for a related feasibility study using an iterative consultation process with parents, academics, and practitioners (N=46) [41,42]. Messages aim to provide new fathers with information, support them in caring for their own physical and mental health, nurturing strong relationships with their child, and developing strong parenting partnerships with their partner. Messages are timed according the babies expected or actual date of birth and thereby designed to address issues that are likely to be occurring for the father when the information arrives. Messages, including links, are limited to 160 characters and designed to engage with fathers through humor, by use of the baby’s voice, and through an encouraging, nonjudgemental tone. Approximately one third of messages (n=98) contain links to online sources of information directly related to the message content [41].

A father participating in the full 77-week program will receive 294 messages. These are usually delivered on a 4-week cycle (5 messages in week 1, 4 in week 2, 3 in week 3, and 3 in week 4) totalling 15 messages. Some messages address particular issues such as alcohol consumption (n=25) and others link to the Mood Tracker application (n=30). Mood Tracker messages ask fathers to respond to questions about their mood on a 5-point scale. Fathers indicating high levels of distress on Mood Tracker will be automatically linked to appropriate websites and offered the opportunity to be contacted by a phone counselling service experienced in paternal perinatal support.

### Study Measures

Measures will be constrained to minimise the burden on participants. Questionnaires employed in the study include the DASS-21 (21 items) [38], the Alcohol Use Disorders Identification Test (AUDIT-C) (3 items) [40], the Short Version Coparenting Relationships Scale (CRS) (14 items) [43], and the Karitane Parenting Confidence Scale (KPCS) (15 items) [44].

The DASS-21 is a truncated version of a 42-item measure designed to assess three distinct, but empirically related, states of mental health. The validity of the three scales (depression, anxiety, and stress) that make up the DASS-21 has been demonstrated through strong correlations with established measures of each factor in diverse populations. The reliability of the subscales has been established in a large normative sample (N=717) with correlations between subscales and relevant measures reported for depression (0.81), anxiety (0.73), and stress (0.81) [39].

The AUDIT-C is a 3-item measure of alcohol consumption. The AUDIT-C was found to be more reliable than telephone interview in detecting heavy drinking in a sample (N=243) of known drinkers [45].

The CRS short version is a truncated version of the 35-item multi-domain questionnaire designed to assess parent perceptions of the strength of their parenting relationship with their partner. The validity of the CRS has been determined against established scales of coparenting quality and the total scale reliability (Cronbach's alpha) across three waves of measurements ranging from 0.81 to 0.85 [43].

The KPCS is designed to assess maternal and paternal perceptions of parenting self-efficacy (PSE) in the Australian context. The validity of the KPCS has been demonstrated against established measures of PSE and the authors report a total scale reliability (Cronbach's alpha) of 0.81 in a normative sample (N=27) [44].

### Data Analyses

Given that there are multiple measures of the primary outcome, ie, DASS-21 scores at baseline and 24 weeks, we will use a linear mixed–model to analyse the results. This model will handle repeated measures as well as missing data; the model will include terms for group, time, and group x time interaction, which will allow us to see if the effect is more marked in one group versus another at particular time points. Secondary outcomes such as parenting, coparenting, and alcohol scales will be analysed using similar models.

## Results

Participant recruitment commenced in October 2016 and will continue until the September 31, 2017. Follow-up data collection has commenced and will be completed March 2018 with results expected in June 2018.

## Discussion

Fathers’ depression and distress can have serious impacts on the wellbeing of their partner and the healthy development of their infants [4,5,11]. The costs of paternal depression are also significant [18]. Yet, fathers are unlikely to seek support with their new role and may not recognize when they need help with their depressed mood or anxiety [10].

Mobile phones offer a flexible, accessible, and cost-effective channel to provide information that can enhance perinatal well-being in fathers and their families. The SMS4dads project has developed an innovative program with a unique combination of direct communication, linked to online resources and telephone support. The message content, which has been developed with extensive review by new parents and experts from the field of perinatal mental health, addresses fathers’ relationship with his infant, his relationship with his partner, and his self-care. The Mood Tracker component provides a safety net for fathers who are not coping and who may benefit from direct telephone contact.

The lack of intrusiveness, high acceptability, and low cost per user indicates the potential to scale up SMS4dads to large numbers of fathers. This study will be the first perinatal trial to assess the efficacy of direct text support for men in their transition to fatherhood. As such, this project may provide guidance for policy development and health care practice across Australia.

## Appendix

Multimedia Appendix 1 Fletcher 2017 SMS4dads slides for presentations.

Multimedia Appendix 2 SMS4dads brief promotion video.

Multimedia Appendix 3 Mood tracker.

## Abbreviations

API: Application programming interface
AUDIT-C: Alcohol Use Disorders Identification Test
CRS: Coparenting Relationships Scale
DASS-21: Depression Anxiety Stress Scales
KPCS: Karitane Parenting Confidence Scale
PPND: Paternal Perinatal Depression
PSE: parenting self-efficacy
