# Supplementary material for: Text-Based Program Addressing the Mental Health of Soon-to-be and New Fathers (SMS4dads): Protocol for a Randomized Controlled Trial
Source: JMIR Res Protoc. 2018 Feb 6;7(2):e37. doi: 10.2196/resprot.8368 (PMC5820459; doi:10.2196/resprot.8368)

## Slide 1
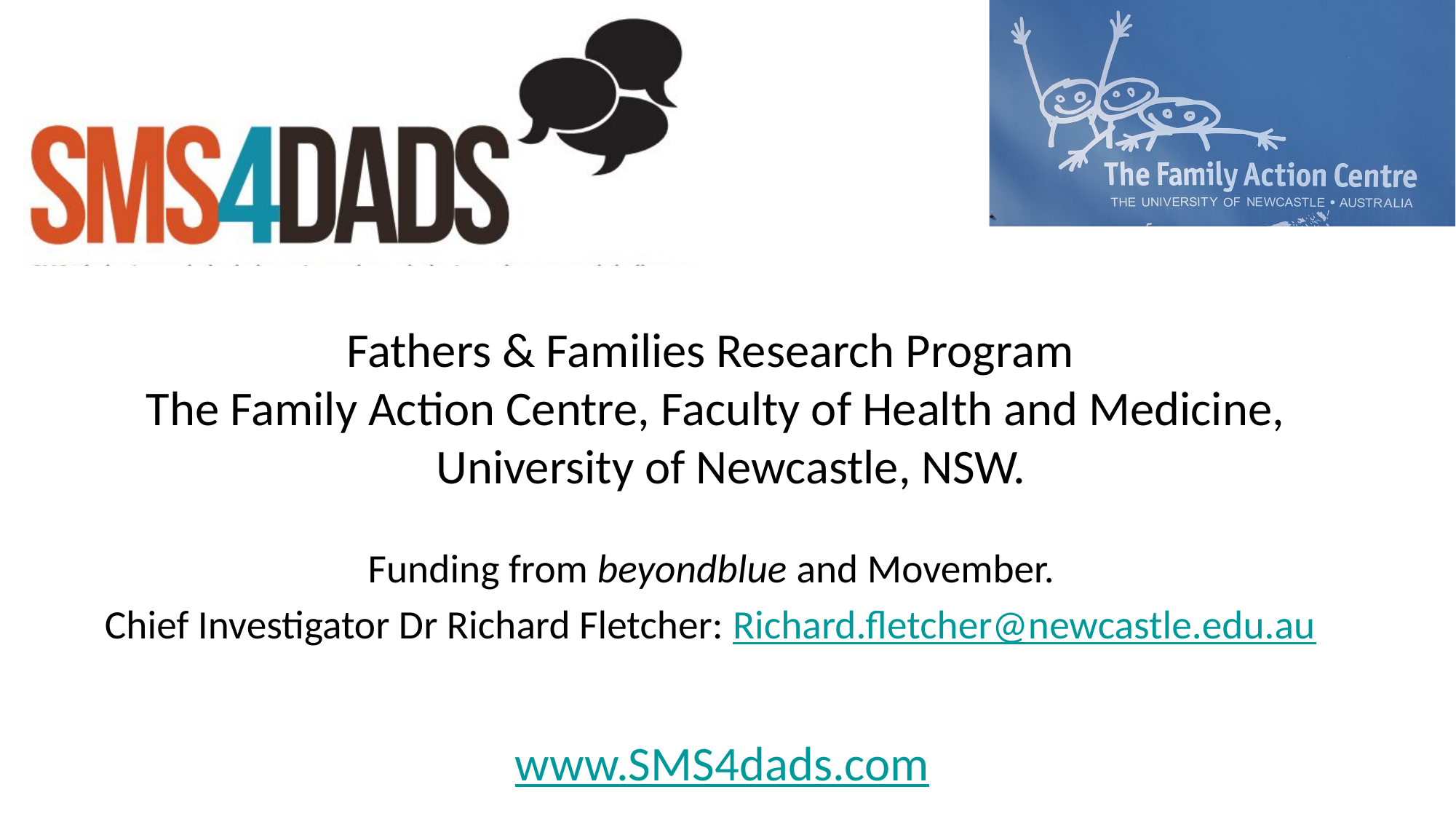

#
Fathers & Families Research Program
The Family Action Centre, Faculty of Health and Medicine, University of Newcastle, NSW.
Funding from beyondblue and Movember.
Chief Investigator Dr Richard Fletcher: Richard.fletcher@newcastle.edu.au
www.SMS4dads.com

## Slide 2
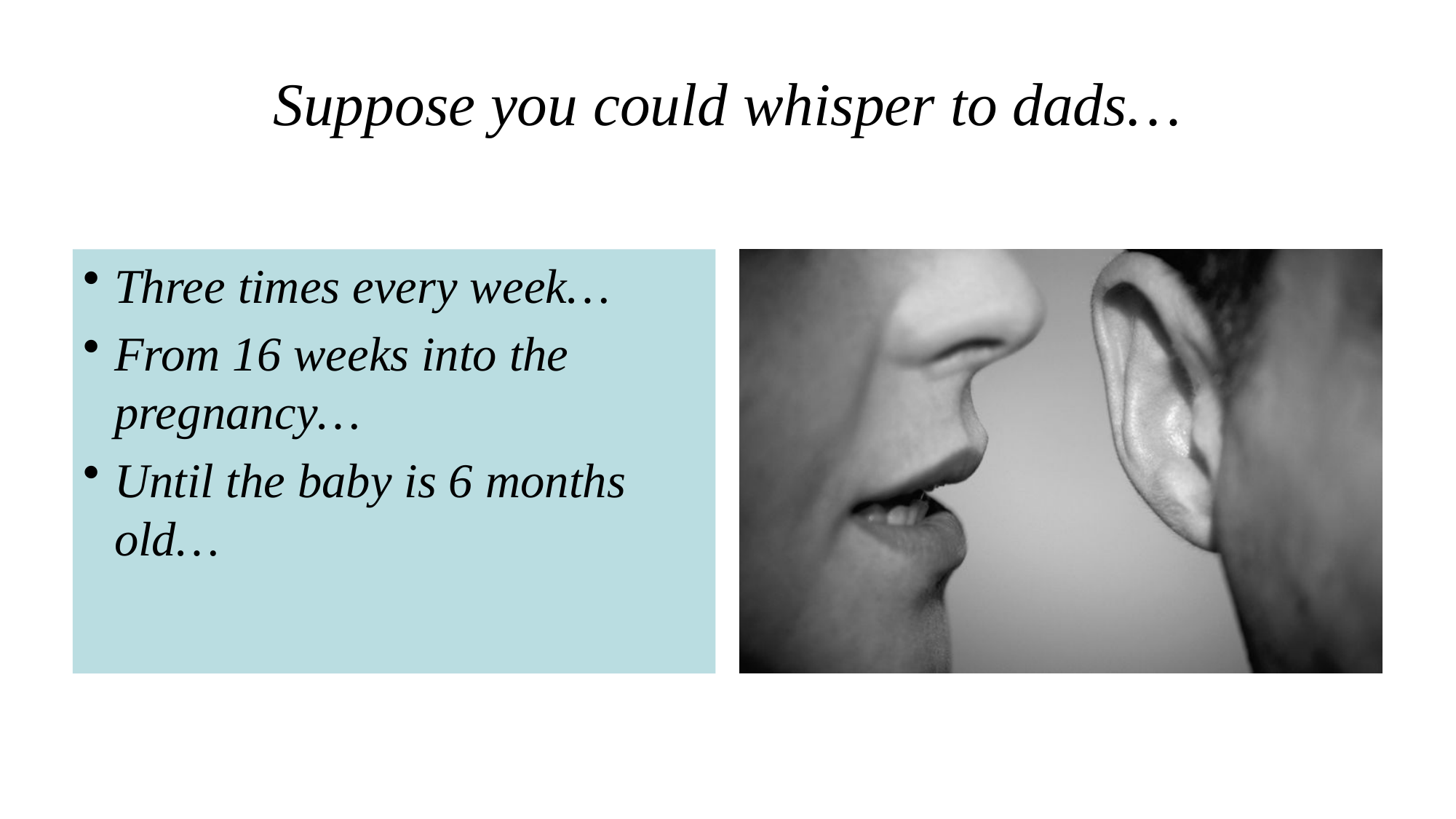

# Suppose you could whisper to dads…
Three times every week…
From 16 weeks into the pregnancy…
Until the baby is 6 months old…

## Slide 3
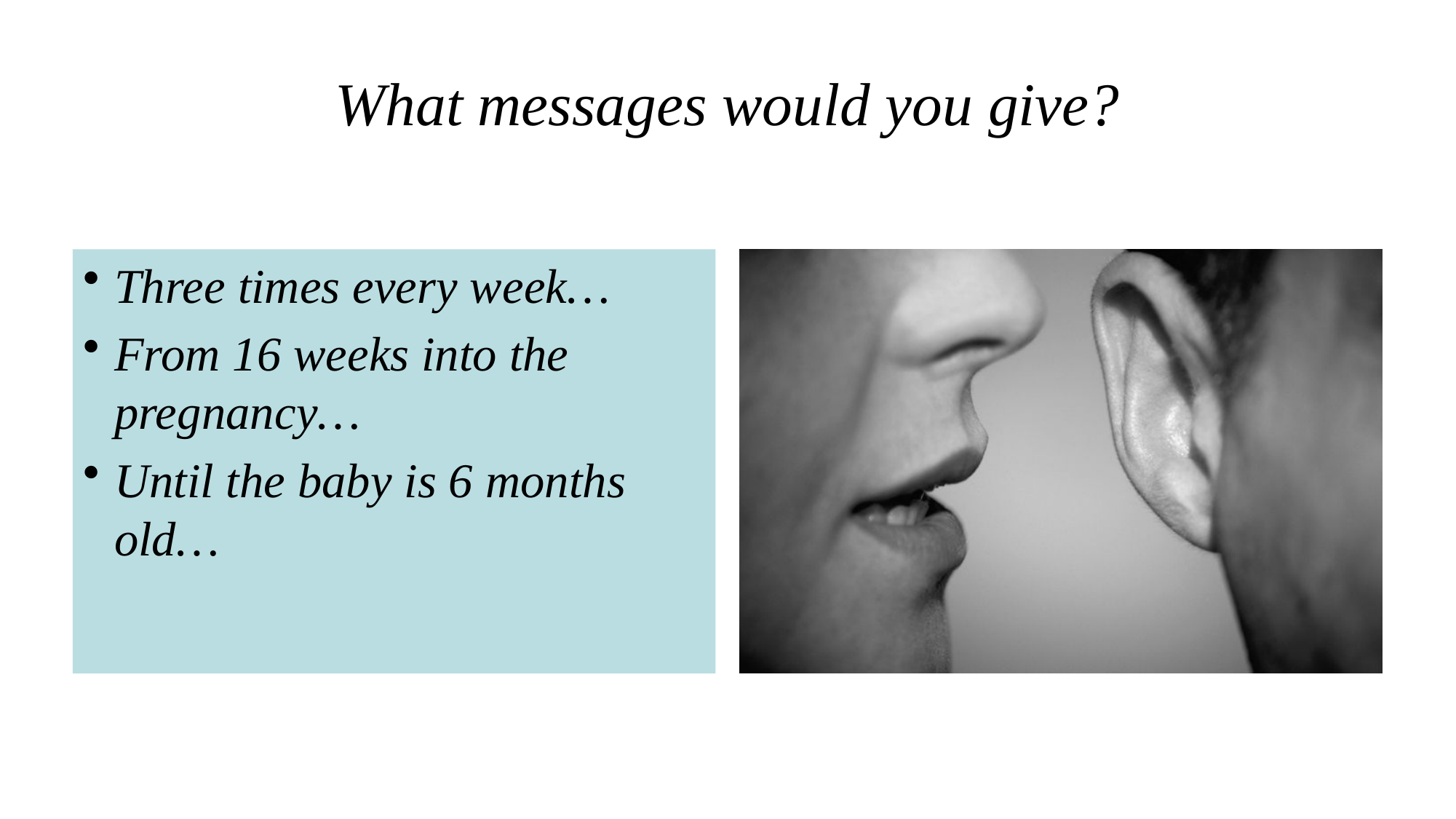

# What messages would you give?
Three times every week…
From 16 weeks into the pregnancy…
Until the baby is 6 months old…

## Slide 4
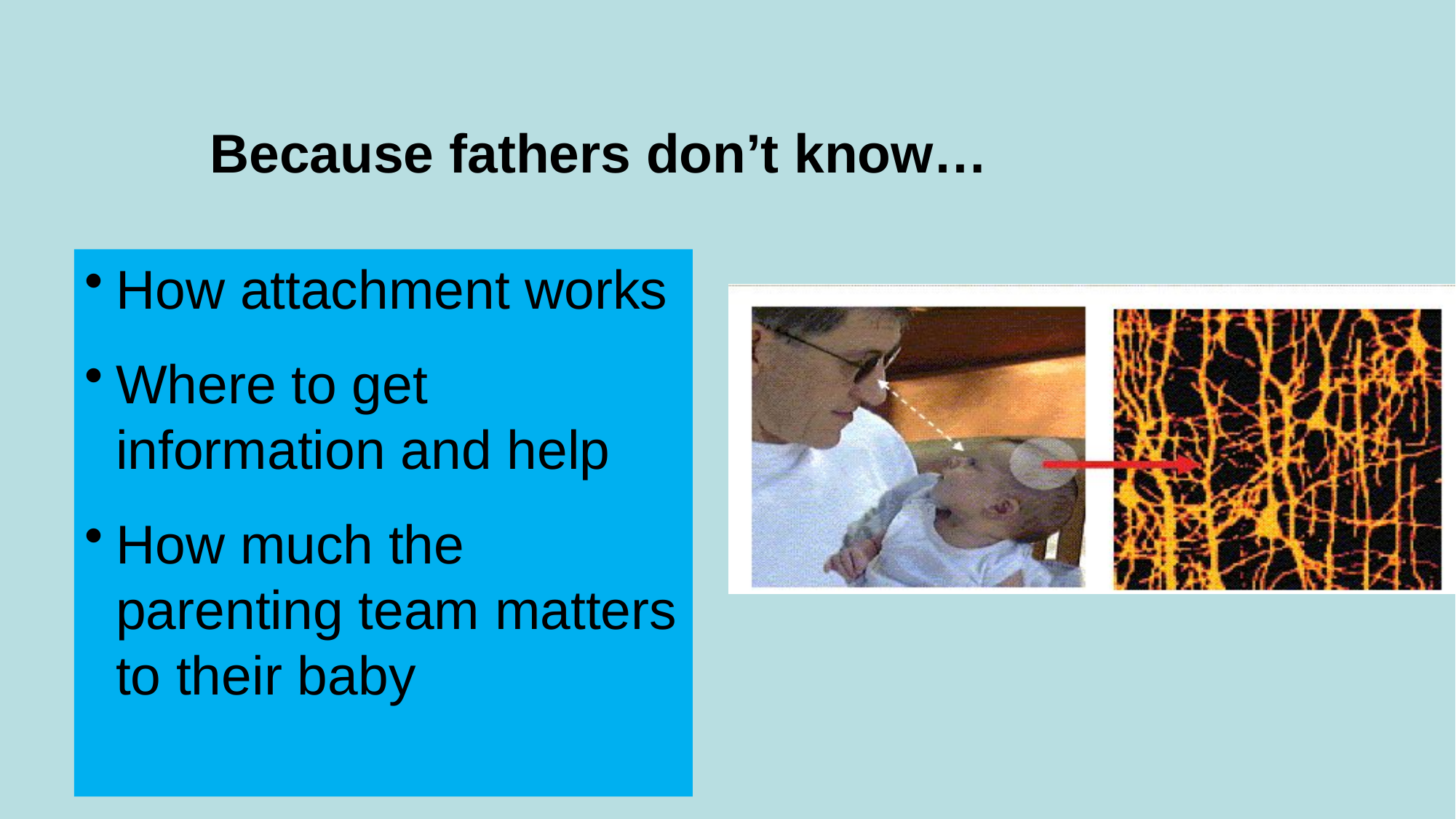

# Because fathers don’t know…
How attachment works
Where to get information and help
How much the parenting team matters to their baby

## Slide 5
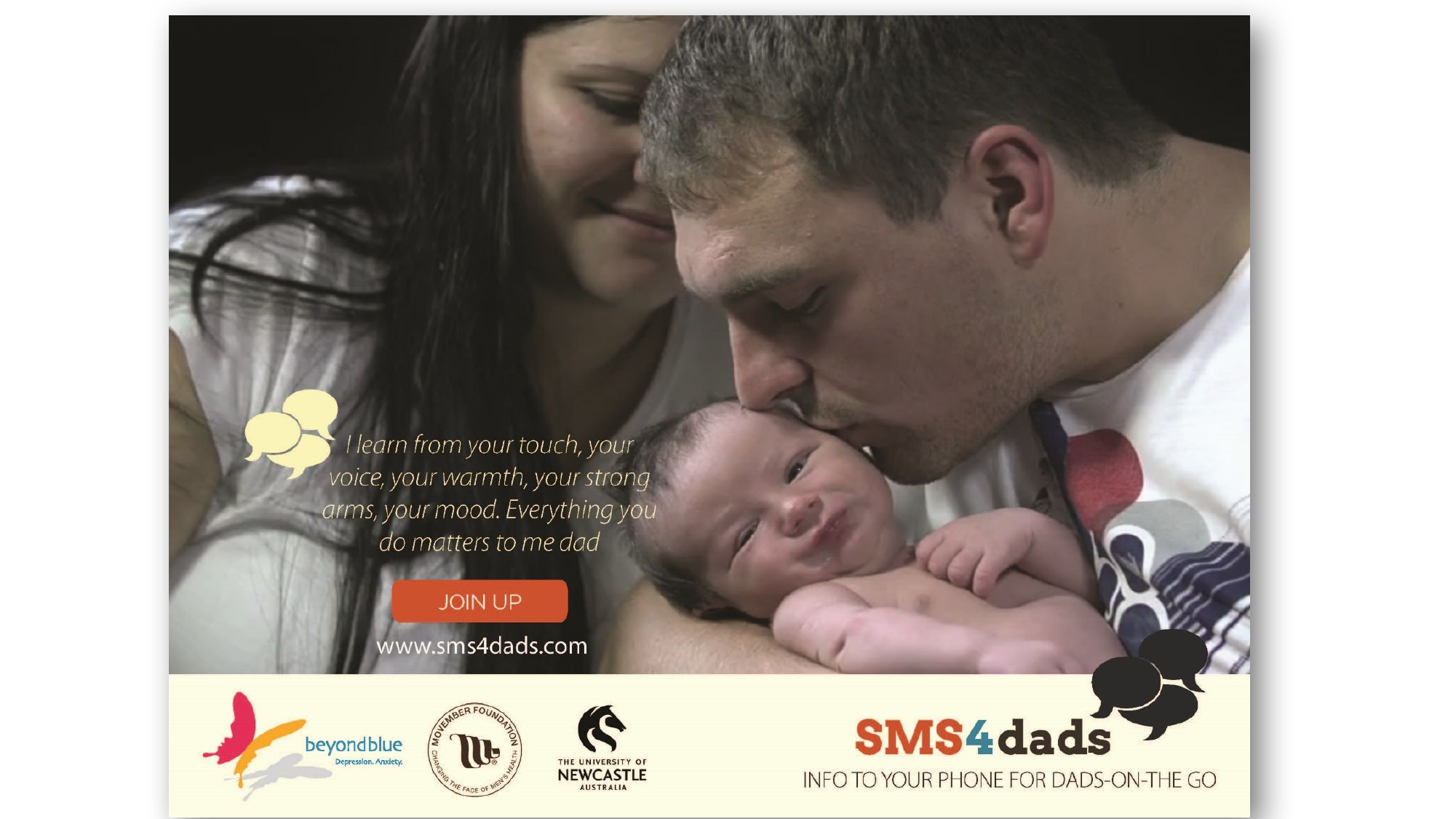

## Slide 6
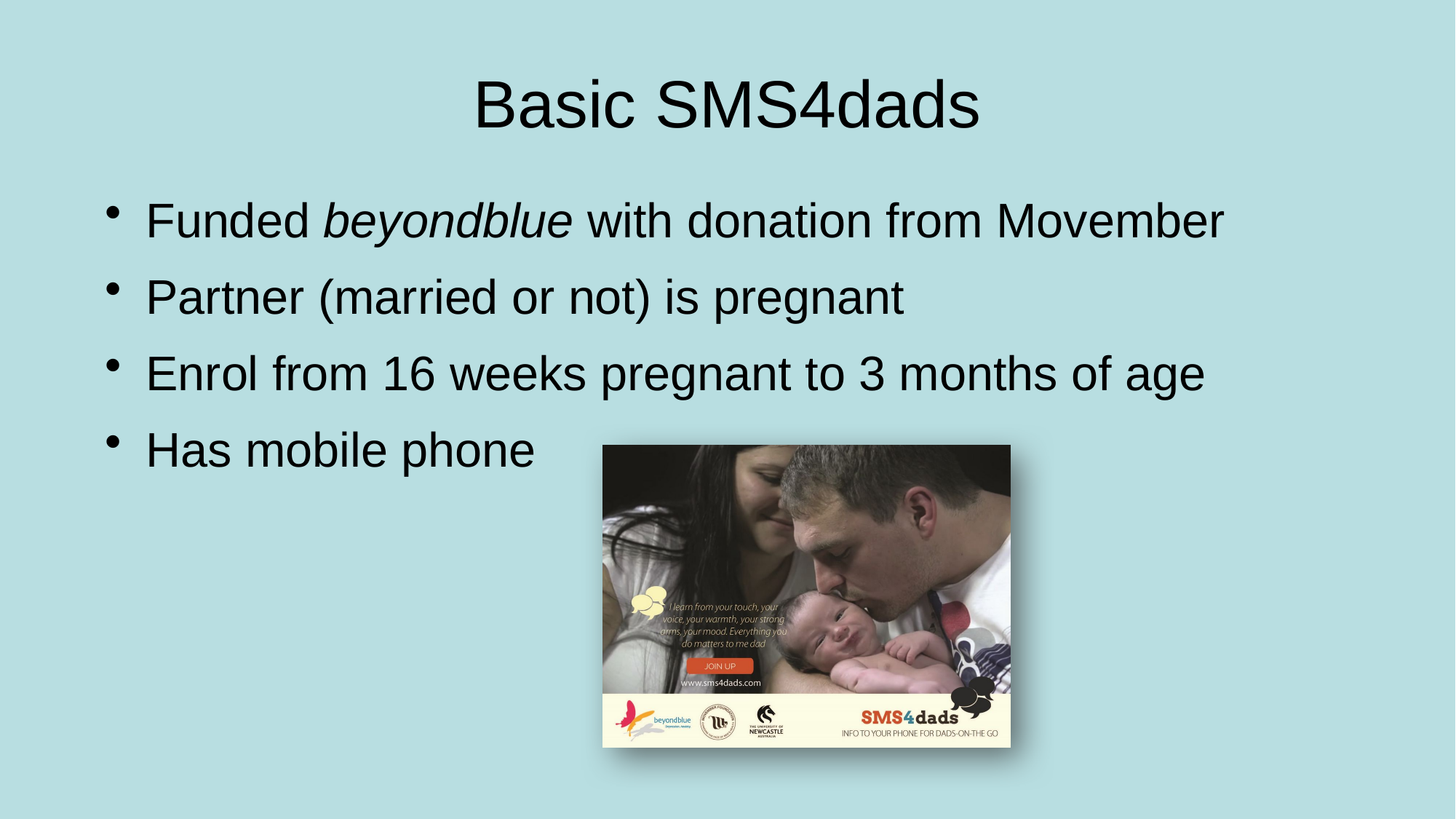

# Basic SMS4dads
Funded beyondblue with donation from Movember
Partner (married or not) is pregnant
Enrol from 16 weeks pregnant to 3 months of age
Has mobile phone

## Slide 7
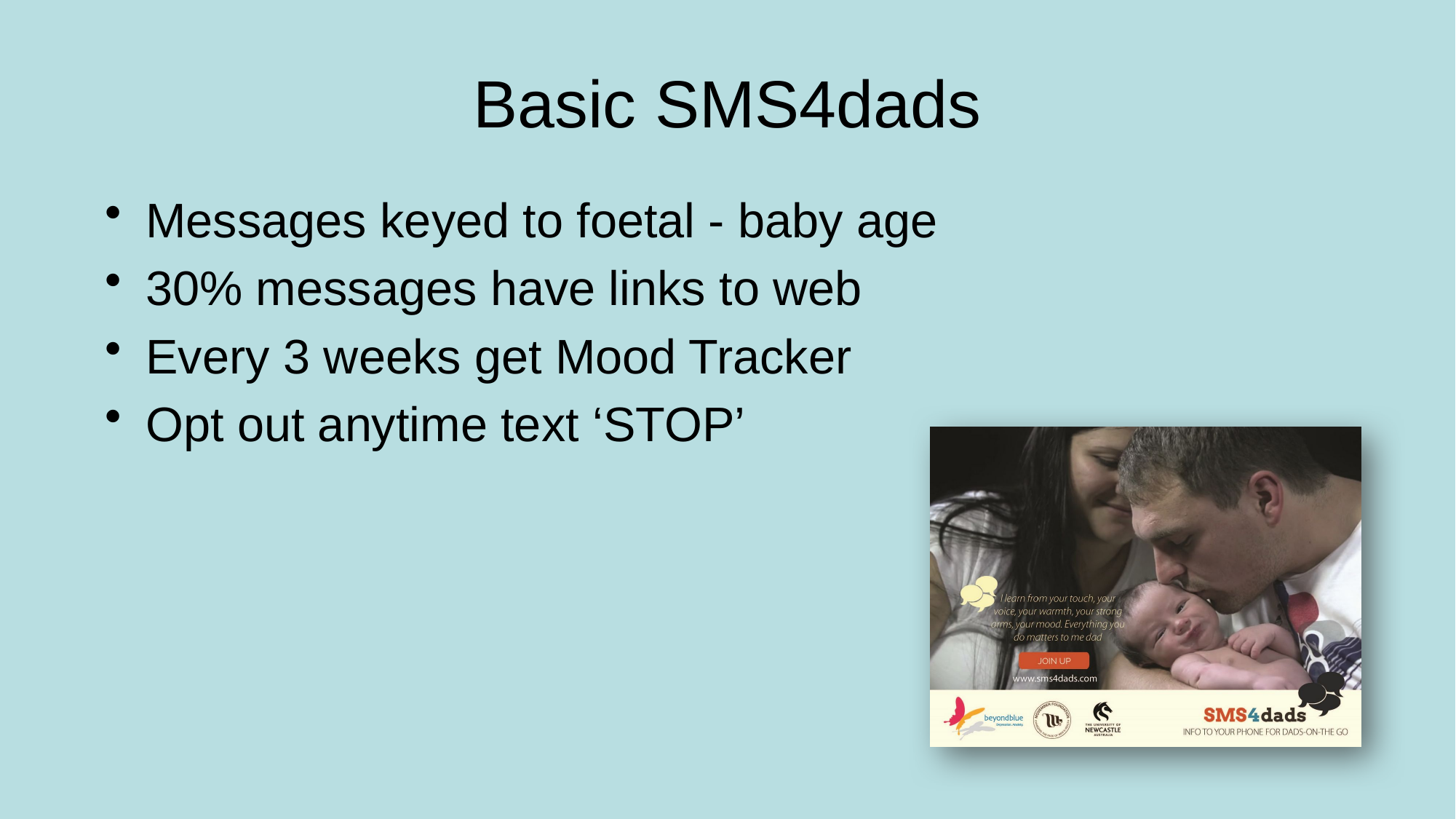

# Basic SMS4dads
Messages keyed to foetal - baby age
30% messages have links to web
Every 3 weeks get Mood Tracker
Opt out anytime text ‘STOP’

## Slide 8
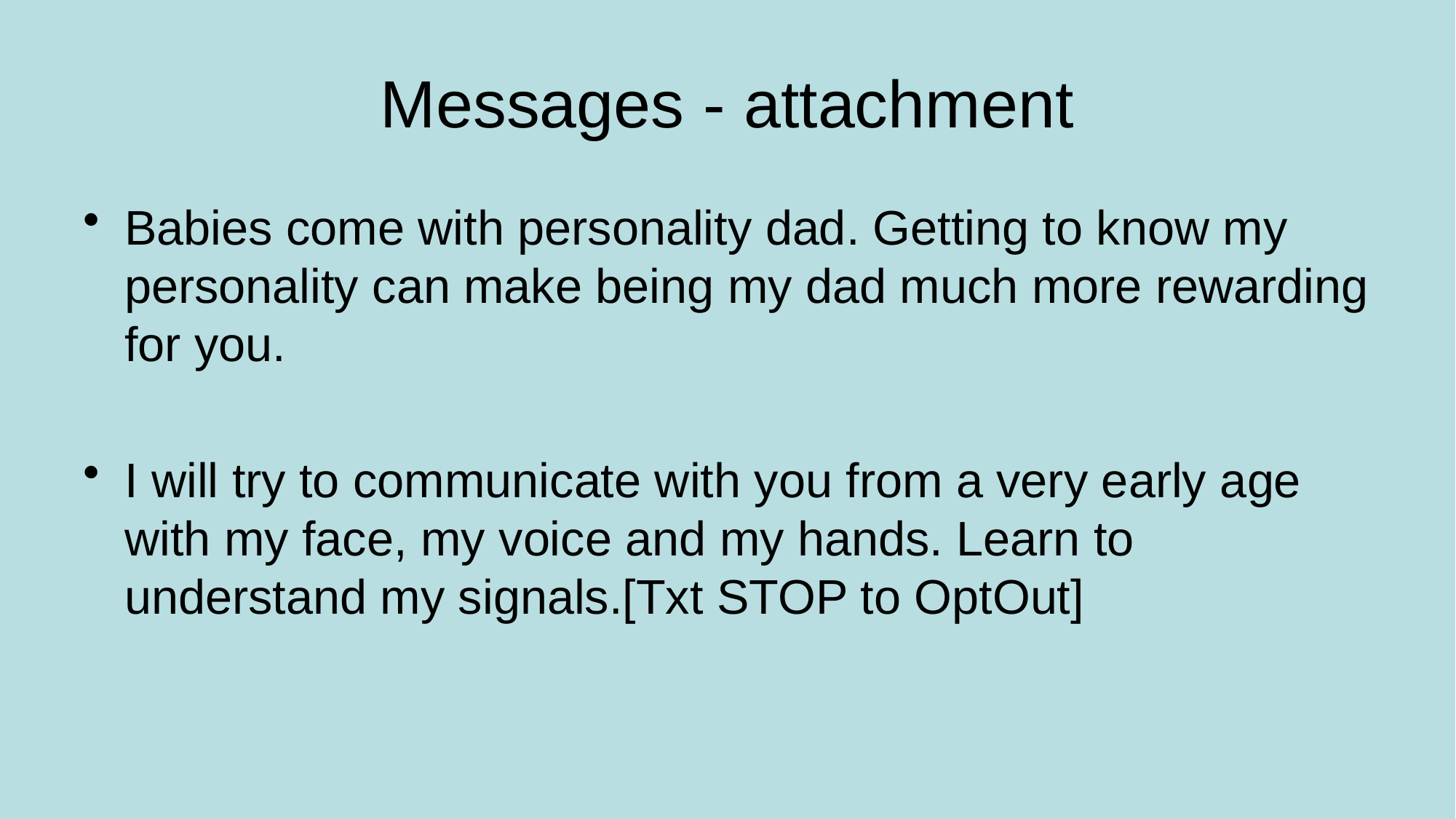

# Messages - attachment
Babies come with personality dad. Getting to know my personality can make being my dad much more rewarding for you.
I will try to communicate with you from a very early age with my face, my voice and my hands. Learn to understand my signals.[Txt STOP to OptOut]

## Slide 9
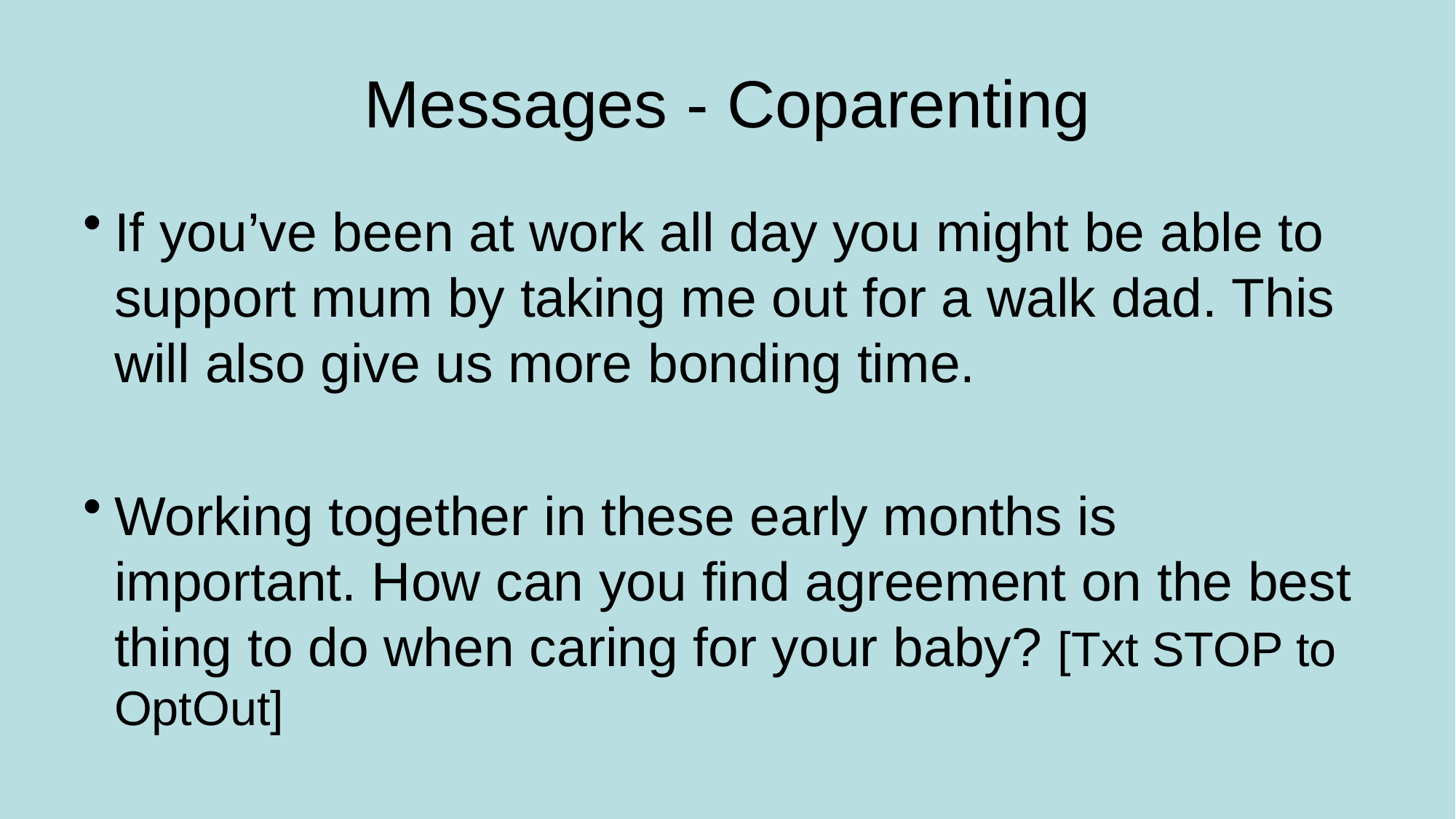

# Messages - Coparenting
If you’ve been at work all day you might be able to support mum by taking me out for a walk dad. This will also give us more bonding time.
Working together in these early months is important. How can you find agreement on the best thing to do when caring for your baby? [Txt STOP to OptOut]

## Slide 10
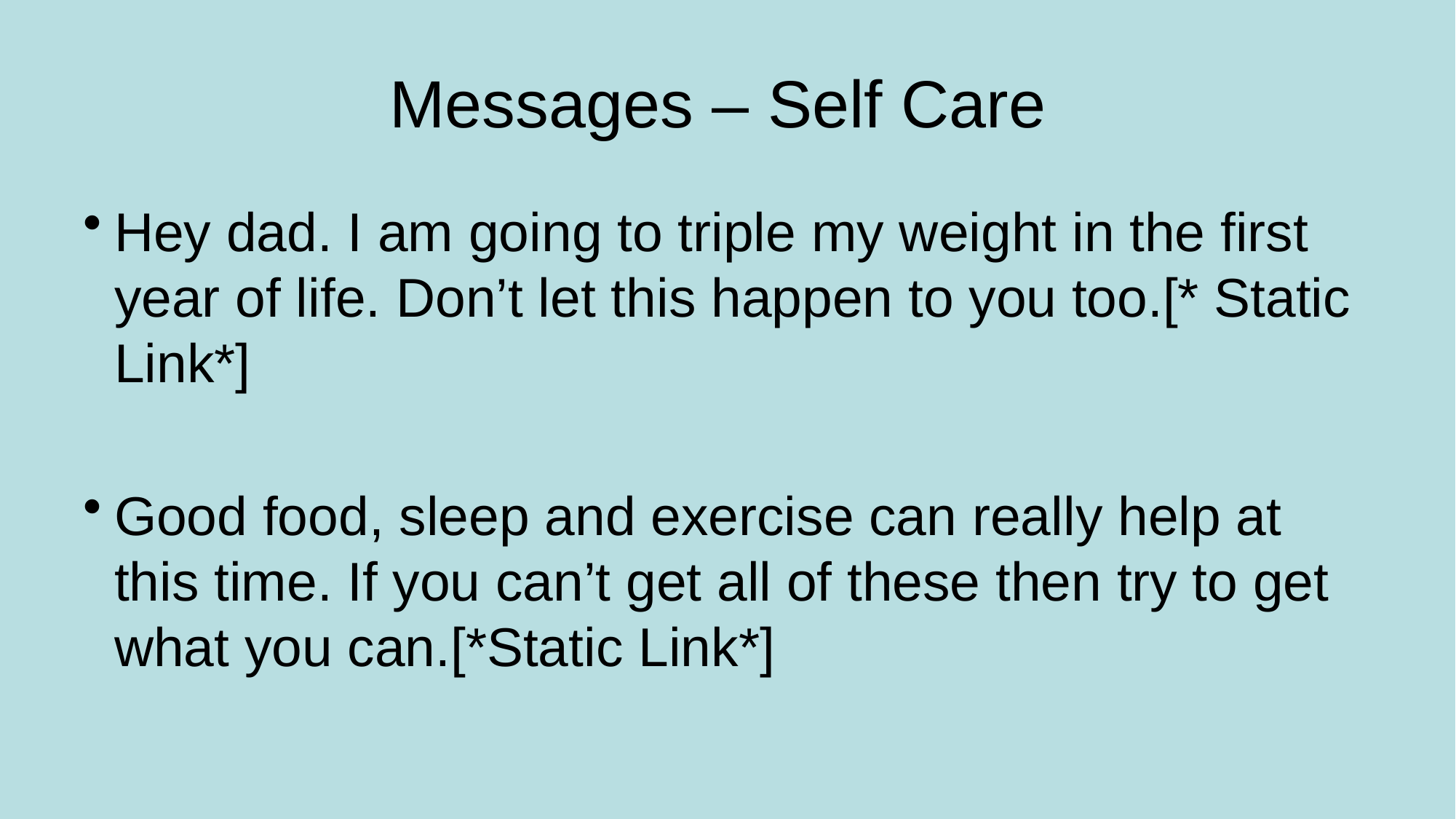

# Messages – Self Care
Hey dad. I am going to triple my weight in the first year of life. Don’t let this happen to you too.[* Static Link*]
Good food, sleep and exercise can really help at this time. If you can’t get all of these then try to get what you can.[*Static Link*]

## Slide 11
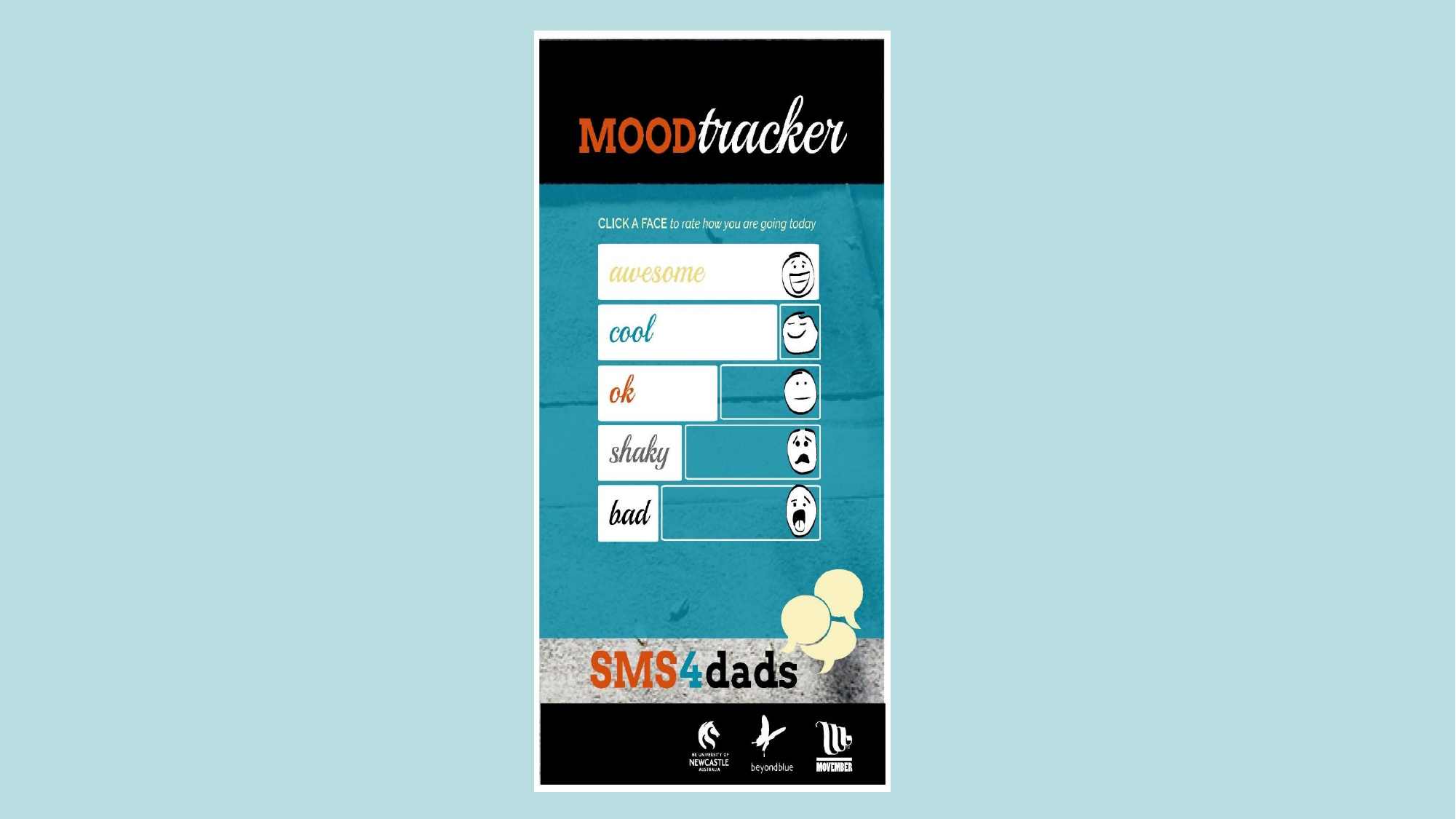

## Slide 12
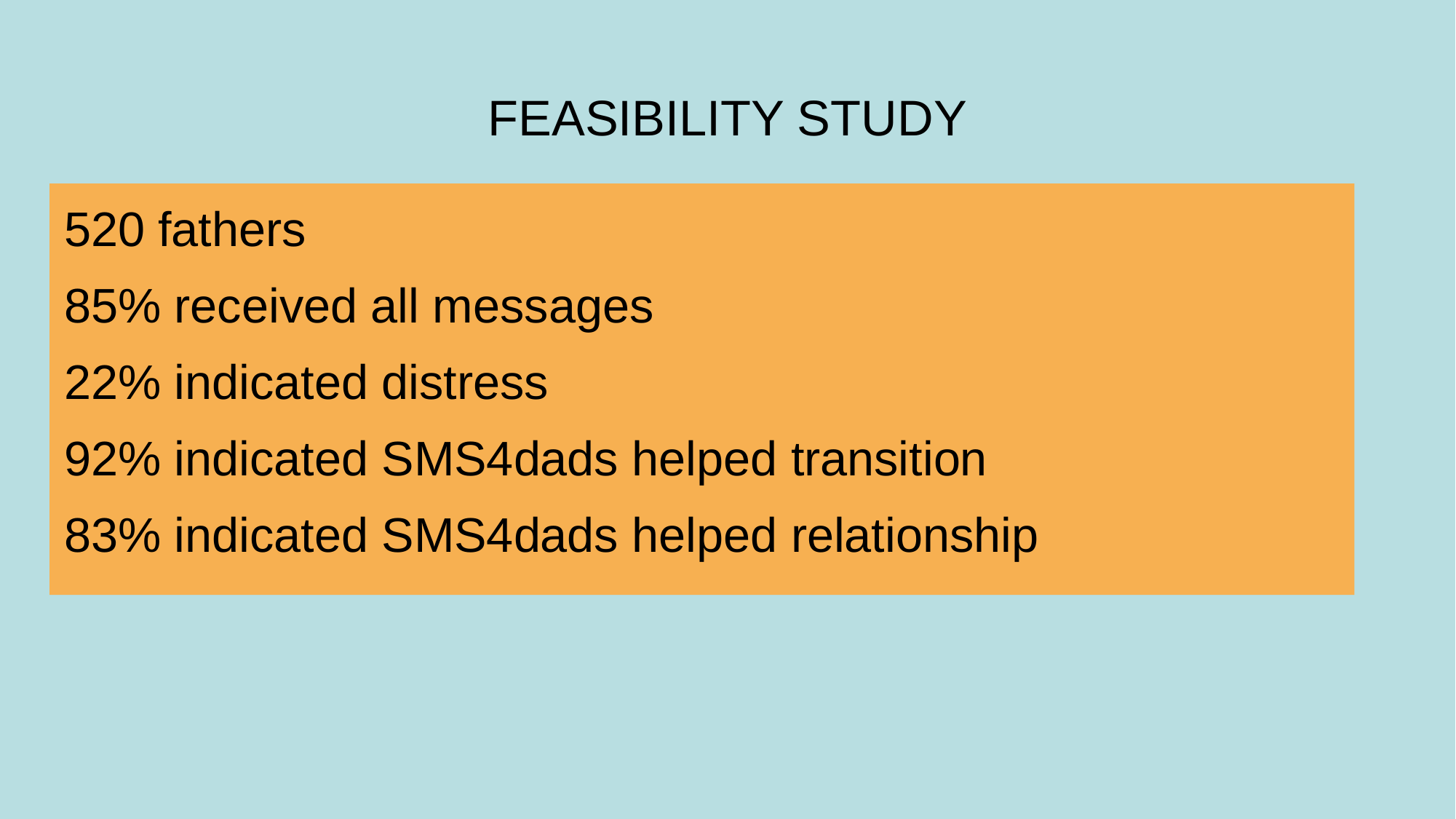

# FEASIBILITY STUDY
520 fathers
85% received all messages
22% indicated distress
92% indicated SMS4dads helped transition
83% indicated SMS4dads helped relationship

## Slide 13
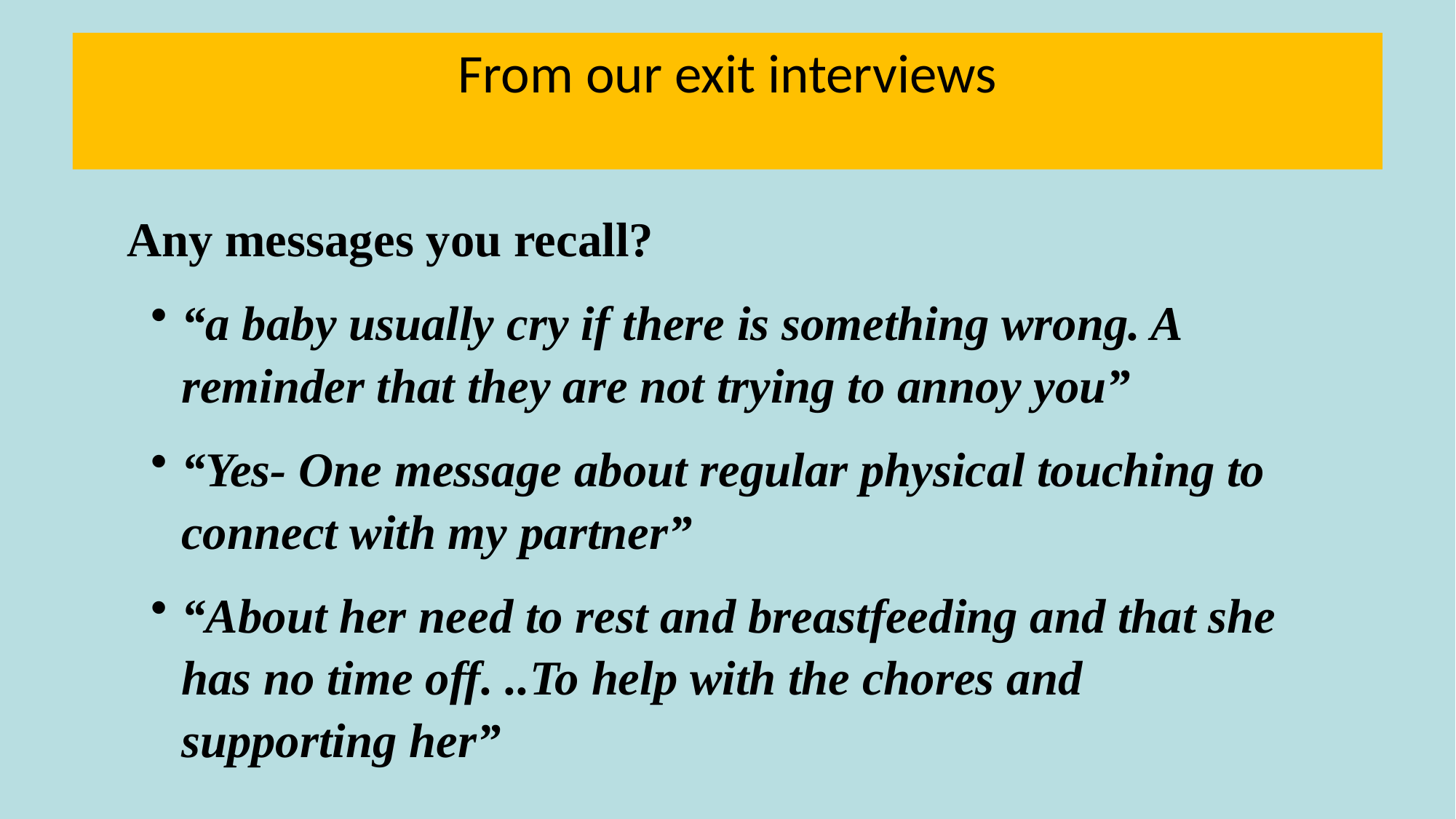

# From our exit interviews
Any messages you recall?
“a baby usually cry if there is something wrong. A reminder that they are not trying to annoy you”
“Yes- One message about regular physical touching to connect with my partner”
“About her need to rest and breastfeeding and that she has no time off. ..To help with the chores and supporting her”

## Slide 14
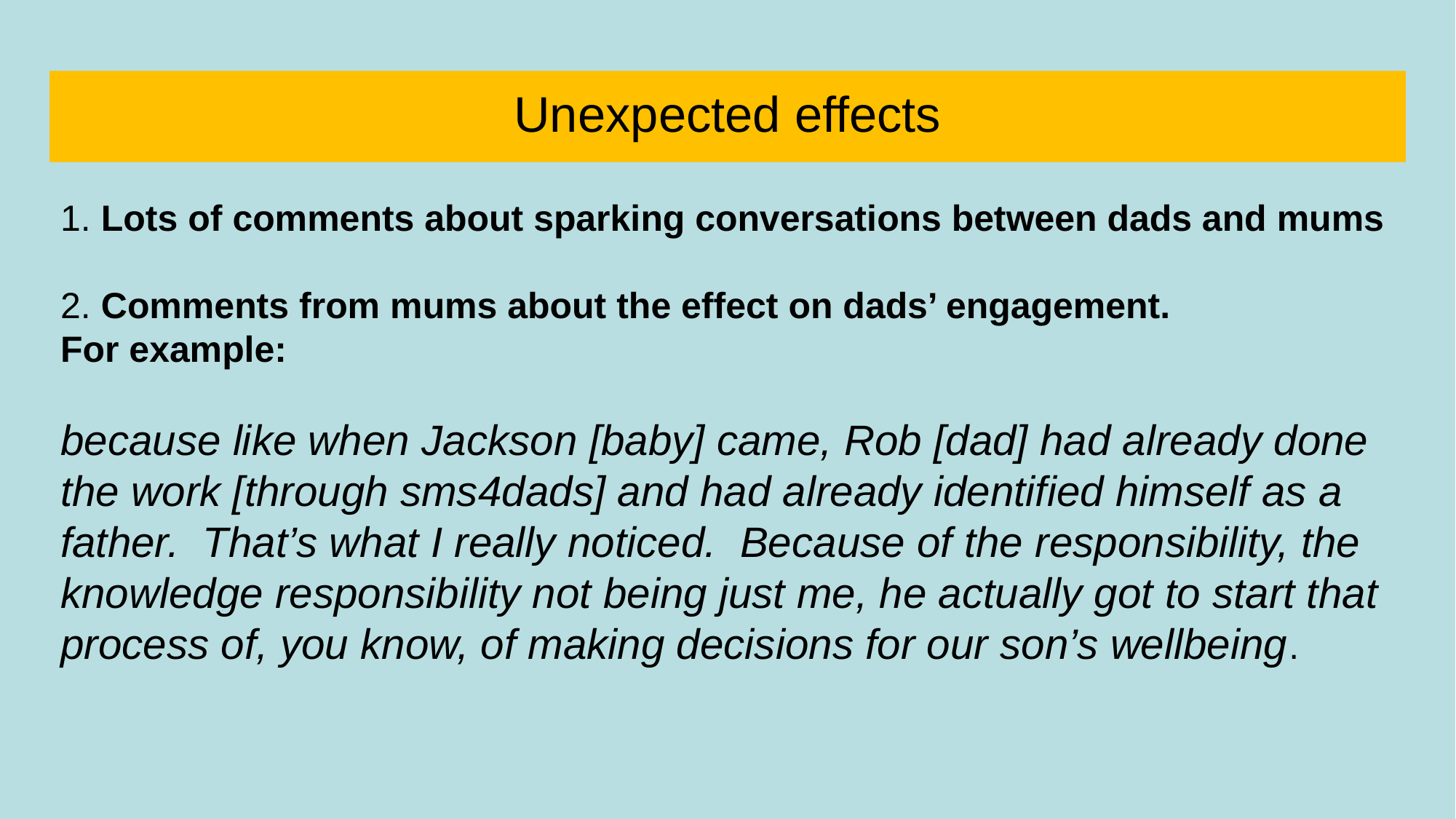

# Unexpected effects
1. Lots of comments about sparking conversations between dads and mums
2. Comments from mums about the effect on dads’ engagement.
For example:
because like when Jackson [baby] came, Rob [dad] had already done the work [through sms4dads] and had already identified himself as a father.  That’s what I really noticed.  Because of the responsibility, the knowledge responsibility not being just me, he actually got to start that process of, you know, of making decisions for our son’s wellbeing.

## Slide 15
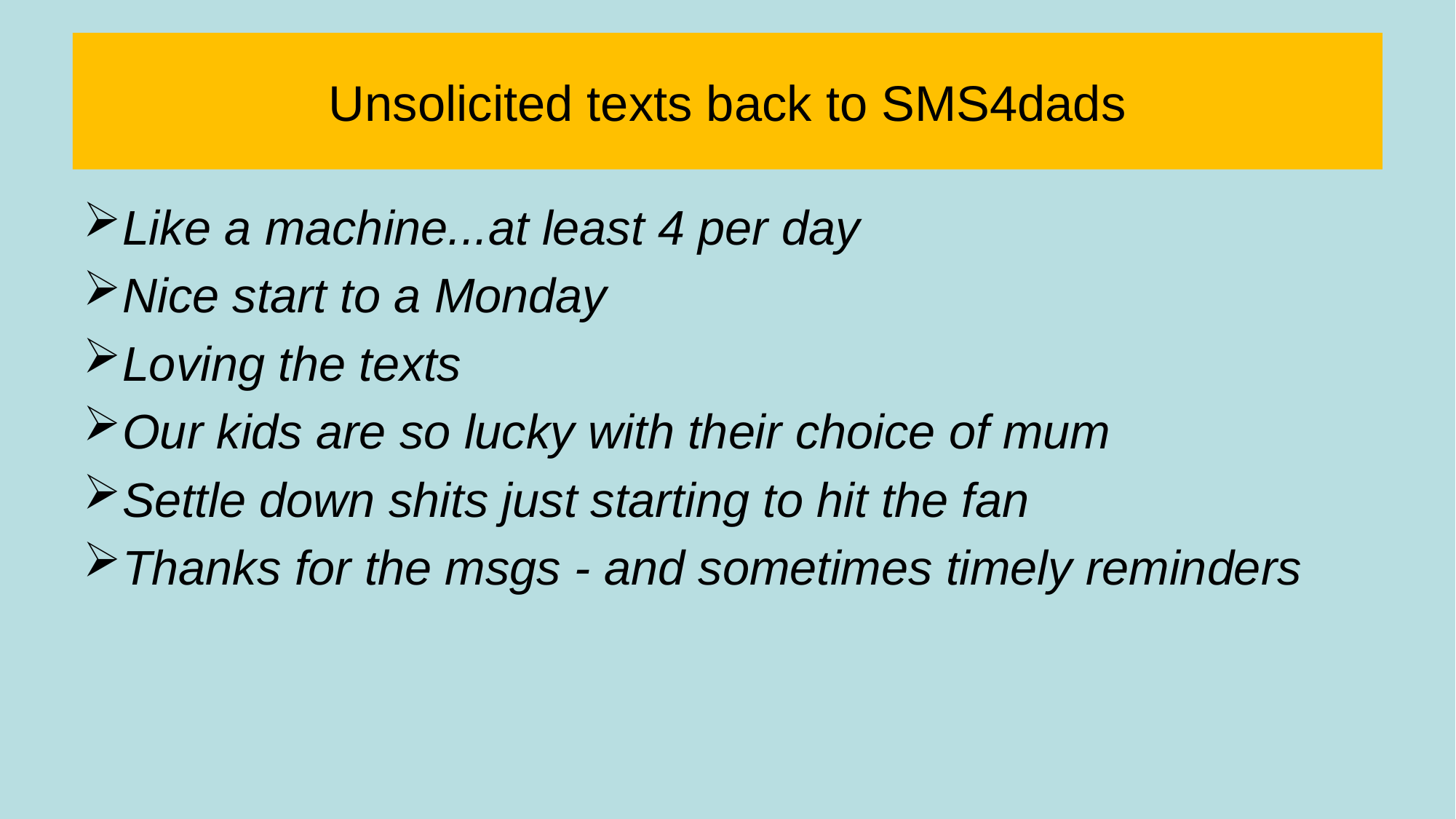

# Unsolicited texts back to SMS4dads
Like a machine...at least 4 per day
Nice start to a Monday
Loving the texts
Our kids are so lucky with their choice of mum
Settle down shits just starting to hit the fan
Thanks for the msgs - and sometimes timely reminders

## Slide 16
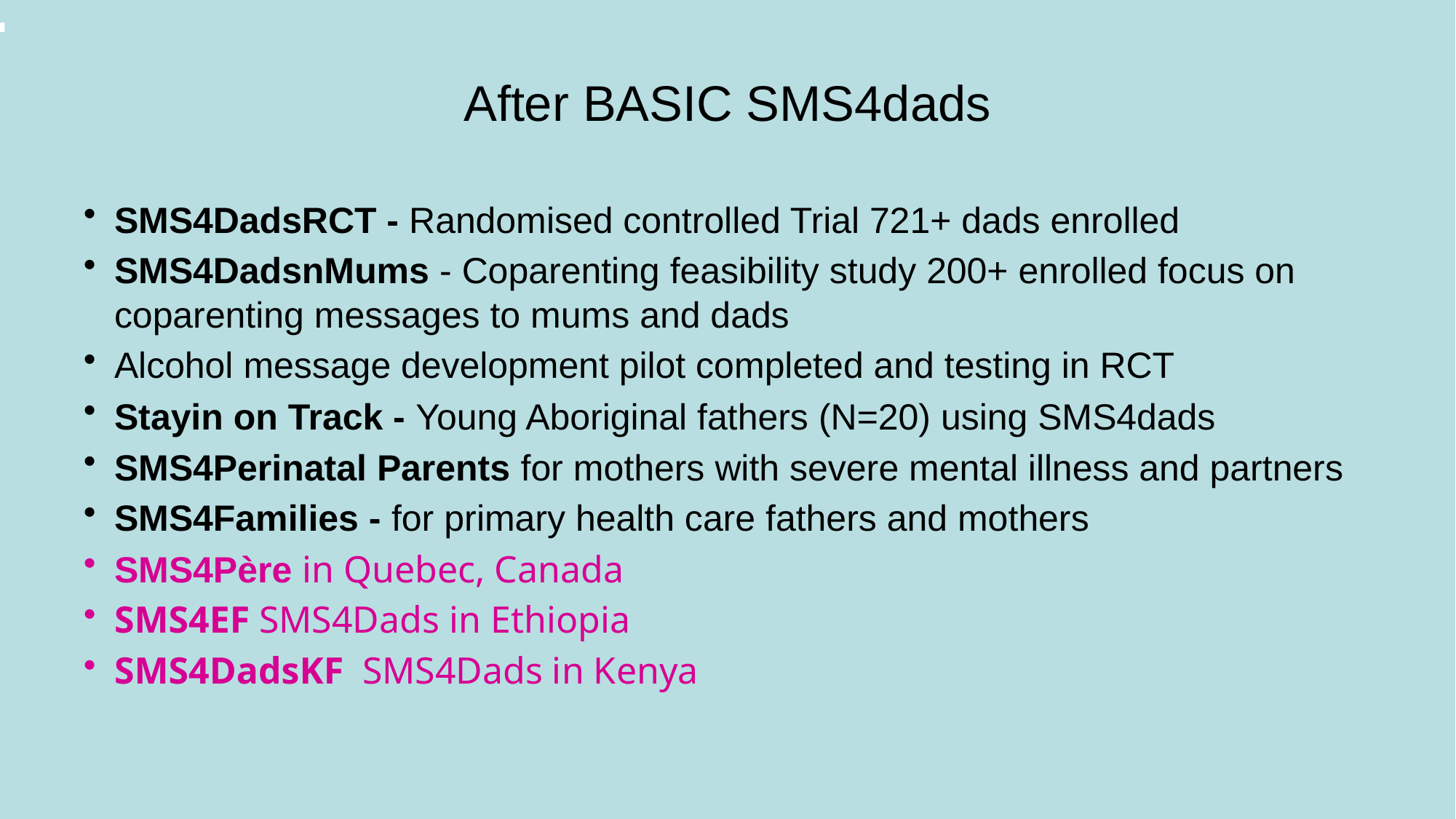

# After BASIC SMS4dads
SMS4DadsRCT - Randomised controlled Trial 721+ dads enrolled
SMS4DadsnMums - Coparenting feasibility study 200+ enrolled focus on coparenting messages to mums and dads
Alcohol message development pilot completed and testing in RCT
Stayin on Track - Young Aboriginal fathers (N=20) using SMS4dads
SMS4Perinatal Parents for mothers with severe mental illness and partners
SMS4Families - for primary health care fathers and mothers
SMS4Père in Quebec, Canada
SMS4EF SMS4Dads in Ethiopia
SMS4DadsKF SMS4Dads in Kenya

## Slide 17
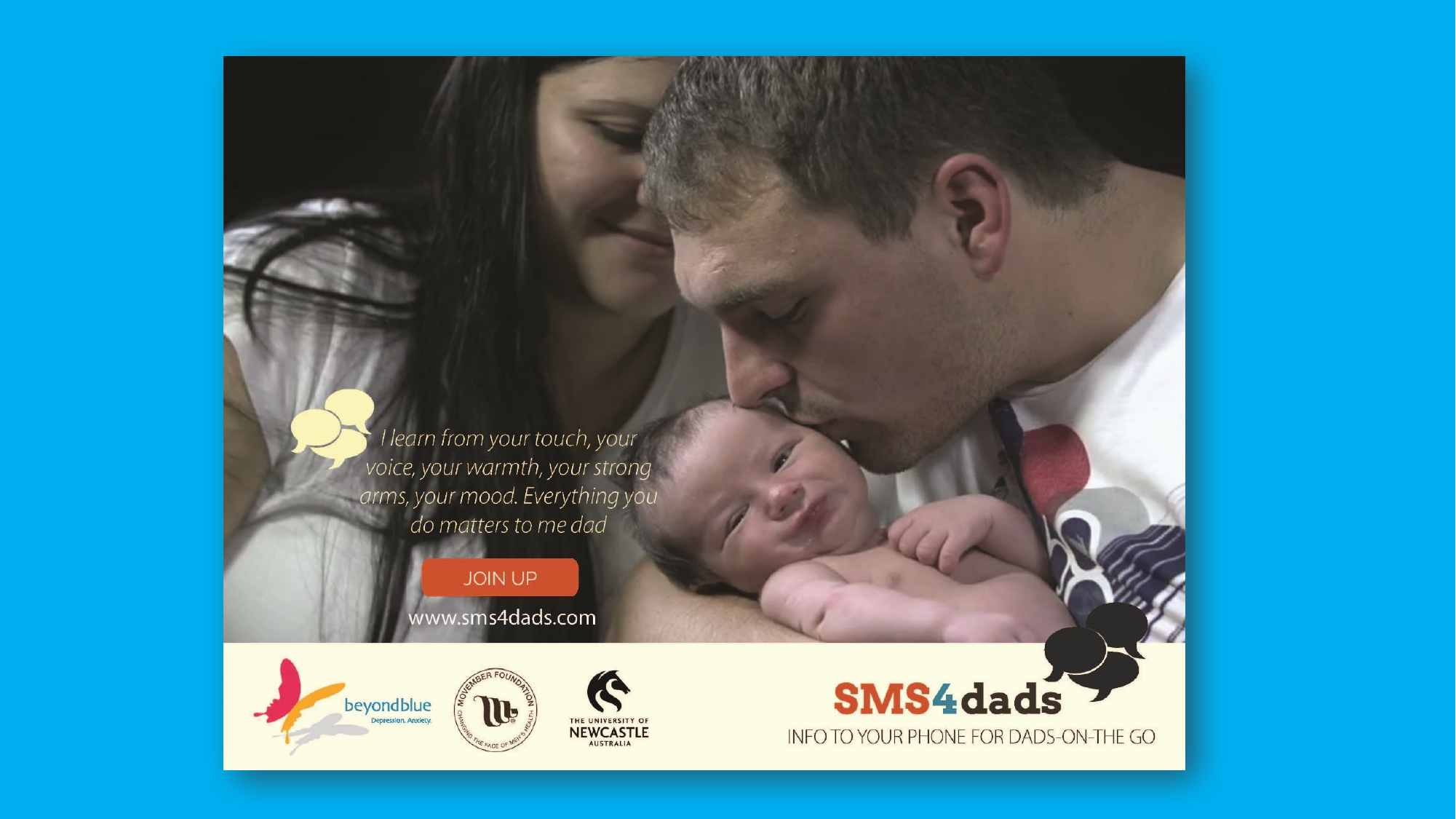

Supplement: Multimedia Appendix 1 [file resprot_v7i2e37_app1.pptx]
