# Supplementary figures and images for: Text-Based Program Addressing the Mental Health of Soon-to-be and New Fathers (SMS4dads): Protocol for a Randomized Controlled Trial
Source: JMIR Res Protoc. 2018 Feb 6;7(2):e37. doi: 10.2196/resprot.8368 (PMC5820459; doi:10.2196/resprot.8368)

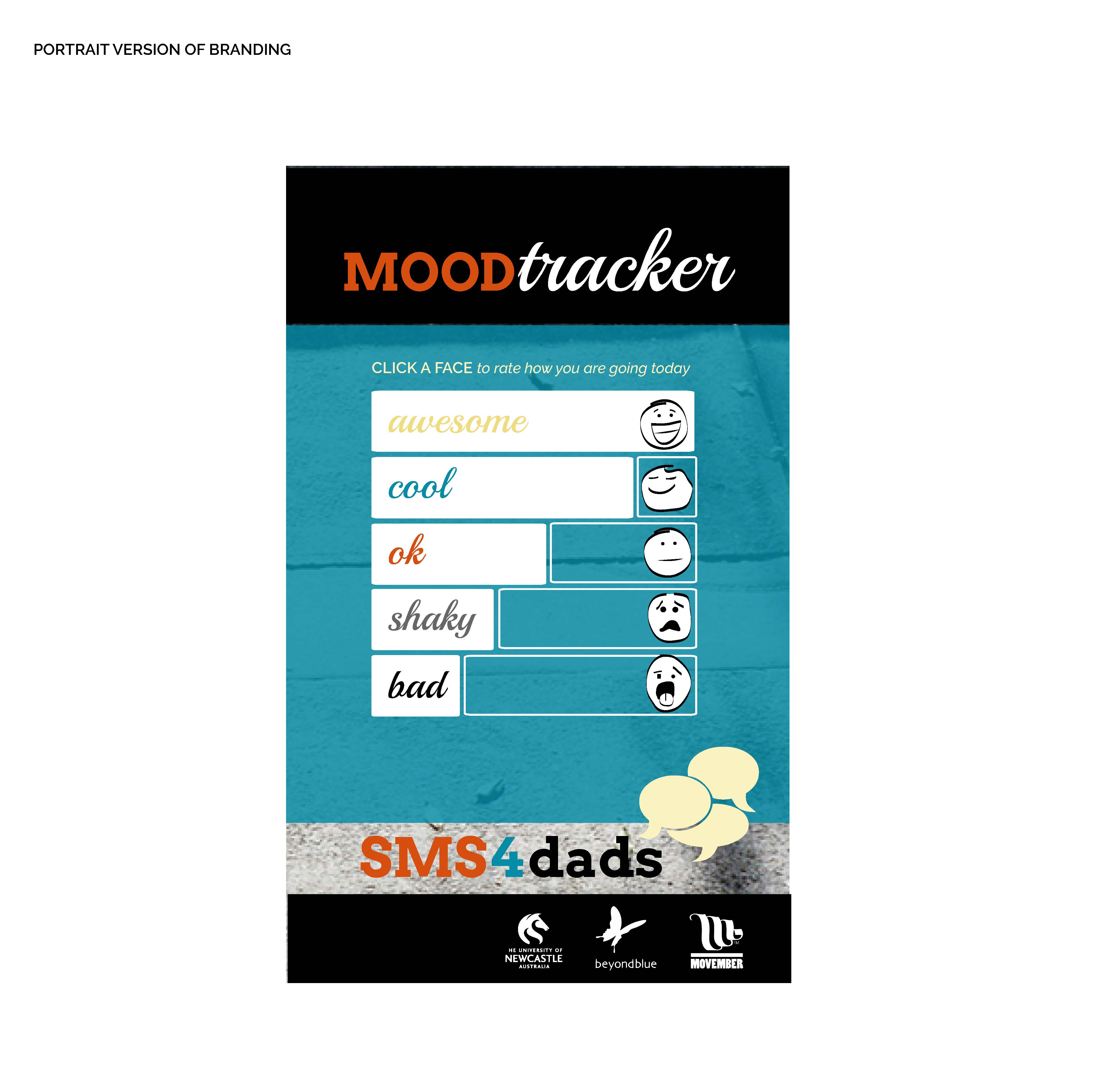

Supplement: Multimedia Appendix 3 [file resprot_v7i2e37_app3.jpg]
